# Computational models in genetics at BGRS\SB-2016: introductory note

**DOI:** 10.1186/s12863-016-0465-3

**Published:** 2016-12-22

**Authors:** Yuriy L. Orlov, Ancha V. Baranova, Arcady L. Markel

**Affiliations:** 1 Institute of Cytology and Genetics SB RAS, Lavrentyeva, 10, 630090 Novosibirsk, Russia; 2 Novosibirsk State University, Pirogova, 2, 630090 Novosibirsk, Russia; 3 School of Systems Biology, George Mason University, Fairfax, VA 22030 USA; 4 Research Centre for Medical Genetics, Moskvorechie 1, Moscow, Russia

This special issue continues the series of BioMed Central special post-conference journal issues (BMC Genetics, BMC Plant biology, BMC Genomics, BMC Evolutionary biology, BMC Systems Biology). All these issues collate the papers presented at BGRS\SB-2016, 10th International Conference “Bioinformatics of Genome Regulation and Structure\Systems Biology” which took place at August 29 - September 2, 2016 in Novosibirsk, Russia. The BGRS conference series started in 1998 in Novosibirsk Akademgorodok (http://conf.bionet.nsc.ru/bgrssb2016/archive/) Since then BGRS/SB was been organized biannually by the Institute of Cytology and Genetics of Siberian Branch of the Russian Academy of Sciences (ICG SB RAS). Across all these years, the Conference was chaired by Prof. Nikolay A. Kolchanov (ICG SB RAS, Russia) and Prof. Ralf Hofestädt (Bielefeld University, Germany). In 2016, the multi-conference held parallel events and symposia on systems biology and biomedicine (SBioMed-2016) (http://conf.bionet.nsc.ru/ishg2016/en/), cognitive sciences (http://physiol.ru/csgb2016/), and mathematical modeling in biology (MM-HPC-BBB-2016) (http://conf.bionet.nsc.ru/mm-hpc-bbb-2016/en/). Since 2014, the BGRS Program Committee has collaborated with BioMed Central on full-text thematic issues reflecting the main science achievements of the conference series in past years. Recently BioMed Central had published several special issues based on best materials presented at the conference in BMC Genetics [1] (http://bmcgenet.biomedcentral.com/articles/supplements/volume-16-supplement-1), BMC Genomics (http://bmcgenomics.biomedcentral.com/articles/supplements/volume-15-supplement-12), BMC Evolutionary Biology (http://bmcevolbiol.biomedcentral.com/articles/supplements/volume-15-supplement-1), and BMC Systems biology (http://bmcsystbiol.biomedcentral.com/articles/supplements/volume-9-supplement-2).

Current issue of BMC Genetics represents latest breakthroughs in genetics discussed at the conference and includes applications of high-throughput sequencing and computational biology for genetics studies in humans and in laboratory animal models. At the Institute of Cytology and Genetics SB RAS – the host of the BGRS\SB-2016 multiconference - these types of studies represent one of the most important research themes. Many of these works are done in frame of national and international collaborations, and involve young researchers working on their PhD Thesis projects.

The paper by E.V.Ignatieva and colleagues [2] opens this special issue by endeavor into the functional annotation of genes regulating feeding behavior, which is a complex problem related to obesity and pre-disposition to the associated diseases. The authors proposed a computational approach to annotation by integrating various mined sources, including previously published original research and review articles, GWAS meta-analyses, and OMIM (Online Mendelian Inheritance in Man) data. A compendium comprising more than 500 human genes controlling food behavior is expected to be useful for pathology risk estimation and for design of new pharmacology approaches to treat human obesity.

The work by Matveeva et al. considers regulatory single nucleotide polymorphisms (rSNPs) in the tumour suppressor APC gene. It was shown that both putative promoters of APC (1A and 1B) drive transcription in an in vitro reporter experiment, many SNPs are functionally relevant and allele G of rs79896135 may be associated with the predisposition to colorectal cancer [3].

The article by Kudryavtseva et al. highlights molecular mechanism of hexokinases function in tumorigenesis of human colorectal cancer and melanoma. The authors studied the effect of silencing hexokinase genes (HKI, HK2, and HK3) in colorectal cancer and melanoma cells using short hairpin RNA (shRNA) lentiviral vectors suggesting the HK1 and HK2 genes as the key therapeutic targets for reducing aerobic glycolysis [4].

Two papers by Korbolina et al. [5] and Ryazanova et al. [6] represent the studies on ISIAH and OXYS rat models developed in ICG SB RAS to study the molecular genetics mechanisms of complex human diseases such as stress-sensitive arterial hypertension and Alzheimer disease.

The paper by Elena Korbolina and colleagues [5] describes the genetic architecture of complex traits, particularly age-related neurodegenerative disorders. The backcrossing of Wistar Albino Glaxo (WAG) and OXYS (an inbred prematurely aging rat strain characterized by high sensitivity to oxidative stress) rat strains to generate the congenics strains resulted in two congenic strains with high susceptibility to cataract and retinopathy but with no signs of Alzheimer’s disease-like brain pathology that are specific for OXYS rats. A comparative analysis of previously defined QTLs and congenic chromosomal segments led to identification of candidate genes with a suspected effect on the brain neurodegeneration.

The ISIAH rat strain (Inherited stress-induced arterial hypertension) was developed by selection for high systolic arterial blood pressure (SABP) induced by restraint stress. Earlier studies showed that the ISIAH rats may be considered as a model of the human stress sensitive hypertensive disease with predominant involvement of the neuroendocrine hypothalamic-pituitary-adrenal (HPA) and sympathoadrenal systems in the pathogenesis of the hypertensive state [7].The studies of the genetic background underlying the stress-sensitive hypertension in the ISIAH rats were already published in special issues of BMC Genetics after BGRS\SB-2014 and SBB-2015 events [8–10]. Current paper outlines the results of the gene-expression profiling in renal medulla of the ISIAH rats and reveal the genes which affect renal function and the long-term control of arterial blood pressure are discussed.

The paper by A. Kononov et al. [11] highlights evolutionary aspects of the moths of genus *Dendrolimus* (Lepidoptera: Lasiocampidae), which are among the major pests of coniferous forests worldwide. The study clarifies the taxonomy of the moths of this genus in Eurasia using mitochondrial markers.

Finally, the work by P. Drozdova et al. [12] discusses a reversible phenotypic switch in yeast model at translation stage. This reversible switching had been attributed to a prion termed [ISP+] and, therefore, is fundamentally relevant to protein aggregate pathologies.

All BGRS\SB-2016 Proceedings including “Bioinformatics and Systems Biology of Plants” section are available at the multi-conference web-site: http://www.bionet.nsc.ru/files/2016/conference/BGRS2016.pdf.

Additionally, special issues on bioinformatics were published at the Journal of Bioinformatics and Computational Biology (http://www.worldscientific.com/toc/jbcb/13/01) [13] and “Vavilov Journal of Selection and Breeding” (http://vavilov.elpub.ru/jour/issue/view/15/showToc) (in Russian).

As usual, BGRS/SB-2016 was accompanied by a number of satellite events, including already traditional Young Scientists School “Systems Biology and Bioinformatics” (SBB-2016) (http://conf.bionet.nsc.ru/sbb2016/en/) and Open Russian-German workshop on bioinformatics network “Systems computational biology”.

## References

[1] Baranova AV, Orlov YL (2016). The papers presented at 7th Young Scientists School “Systems Biology and Bioinformatics” (SBB’15): Introductory Note. BMC Genet.

[2] Ignatieva EV, Afonnikov DA, Saik OV, Rogaev EI, Kolchanov NA. A compendium of human genes regulating feeding behavior and body weight, its functional characterization and identification of GWAS genes involved in brain-specific PPI network. BMC Genet. 2016;17 Suppl 3:2. doi:10.1186/s12863-016-0466-2. 10.1186/s12863-016-0466-2 PMC5249002 28105929

[3] Matveeva MY, Kashina EV, Reshetnikov VV, Bryzgalov LO, Antontseva EV, Bondar NP, Merkulova TI. Regulatory single nucleotide polymorphisms (rSNPs) at the promoters 1A and 1B of the human APC gene. BMC Genet. 2016;17 Suppl 3:3. doi:10.1186/s12863-016-0460-8. 10.1186/s12863-016-0460-8 PMC5249005 28105931

[4] Kudryavtseva AV, Fedorova MS, Dmitriev AA, Zaretsky AR, Afremova A, Kochetkov D, Lipatova AV, Sadritdinova AF, Nasedkina NV, Zasedatelev AS, Alekseev BY, Popov AY, Krasnov GS, Snezhkina AV. Effect of lentivirus-mediated shRNA inactivation of HK1, HK2, and HK3 genes in colorectal cancer and melanoma cells. BMC Genet. 2016;17 Suppl 3:4. doi:10.1186/s12863-016-0459-1. 10.1186/s12863-016-0459-1 PMC5249010 28105937

[5] Korbolina EE, Zhdankina AA, Fursova AZ, Kozhevnikova OS, Kolosova NG. Genes of susceptibility to early neurodegenerative changes in the rat retina and brain: Analysis by means of congenic strains. BMC Genet. 2016;17 Suppl 3:5. doi:10.1186/s12863-016-0461-7. 10.1186/s12863-016-0461-7 PMC5249004 28105932

[6] Ryazanova MA, Fedoseeva LA, Ershov NI, Markel AL, Redina OE. The gene-expression profile of renal medulla in rats with inherited stress-induced arterial hypertension. BMC Genet. 2016;17 Suppl 3:6. doi:10.1186/s12863-016-0462-6. 10.1186/s12863-016-0462-6 PMC5249016 28105926

[7] Markel AL, Redina OE, Gilinsky MA, Dymshits GM, Kalashnikova EV, Khvorostova YV, Fedoseeva LA, Jacobson GS (2007). Neuroendocrine profiling in inherited stress-induced arterial hypertension rat strain with stress-sensitive arterial hypertension. J Endocrinol.

[8] Redina OE, Smolenskaya SE, Klimov LO, Markel AL (2015). Candidate genes in quantitative trait loci associated with absolute and relative kidney weight in rats with Inherited Stress Induced Arterial Hypertension. BMC Genet.

[9] Klimov LO, Ershov NI, Efimov VM, Markel AL, Redina OE (2016). Genome-wide transcriptome analysis of hypothalamus in rats with inherited stress-induced arterial hypertension. BMC Genet.

[10] Fedoseeva LA, Ryazanova MA, Ershov NI, Markel AL, Redina OE (2016). Comparative transcriptional profiling of renal cortex in rats with inherited stress-induced arterial hypertension and normotensive Wistar Albino Glaxo rats. BMC Genet.

[11] Kononov A, Ustyantsev K, Wang B, Mastro VC, Fet V, Blinov AG, Baranchikov Y. Genetic diversity among eight Dendrolimus species in Eurasia (Lepidoptera: Lasiocampidae) inferred from mitochondrial COI and COII, and nuclear ITS2 markers. BMC Genet. 2016;17 Suppl 3:7. doi:10.1186/s12863-016-0463-5. 10.1186/s12863-016-0463-5 PMC5249024 28105930

[12] Drozdova P, Mironova L, Zhouravleva G. Haploid yeast cells undergo a reversible phenotypic switch associated with chromosome II copy number. BMC Genet. 2016;17 Suppl 3:8. doi:10.1186/s12863-016-0464-4. 10.1186/s12863-016-0464-4 PMC5249023 28105933

[13] Orlov YL, Hofestädt RM, Kolchanov NA. Introductory note for BGRS\SB-2014 special issue J. Bioinform. Comput. Biol. 2015; 13, 1502001 DOI: 10.1142/S0219720015020011 10.1142/S0219720015020011 25666651

